# Fabrication and employment of cobalt-doped yttrium iron garnets for the electrochemical analysis of anti-diabetic, metformin in serum of type 2 diabetes mellitus patients

**DOI:** 10.1186/s11671-023-03795-8

**Published:** 2023-02-22

**Authors:** Shan E Zahra Jawad, Muhammad Ibrahim, Batool Fatima, Tahir Ali Chohan, Dilshad Hussain, Muhammad Najam-ul-Haq

**Affiliations:** 1 Department of Biochemistry, Bahauddin Zakariya University, Multan, 60800 Pakistan; 2 Institute of Pharmaceutical Sciences, University of Veterinary and Animal Sciences, Lahore, Pakistan; 3 HEJ Research Institute of Chemistry, International Center for Chemical and Biological Sciences, University of Karachi, Karachi, 75270 Pakistan; 4 Institute of Chemical Sciences, Bahauddin Zakariya University, Multan, 60800 Pakistan

**Keywords:** Type II diabetes mellitus, Metformin, Electrochemical sensor, Cyclic voltammetry, Differential pulse voltammetry, Electrochemical impedance spectroscopy

## Abstract

Metformin (MET) is an anti-diabetic drug employed as the first-line therapy for patients of type II diabetes mellitus (T2DM). Overdosage of drugs leads to severe outcomes, and its monitoring in biofluids is vital. The present study develops cobalt-doped yttrium iron garnets and employs them as an electroactive material immobilized on a glassy carbon electrode (GCE) for the sensitive and selective detection of metformin via electroanalytical techniques. The fabrication procedure via the sol–gel method is facile and gives a good yield of nanoparticles. They are characterized by FTIR, UV, SEM, EDX, and XRD. Pristine yttrium iron garnet particles are also synthesized for comparison, where the electrochemical behaviors of varying electrodes are analyzed via cyclic voltammetry (CV). The activity of metformin at varying concentrations and pH is investigated via differential pulse voltammetry (DPV), and the sensor generates excellent results for metformin detection. Under optimum conditions and at a working potential of 0.85 V (vs. Ag/AgCl/3.0 M KCl), the linear range and limit of detection (LOD) obtained through the calibration curve are estimated as 0–60 μM and 0.04 μM, respectively. The fabricated sensor is selective for metformin and depicts a blind response toward interfering species. The optimized system is applied to directly measure MET in buffers and serum samples of T2DM patients.

## Introduction

Diabetes mellitus (DM) accounts for 90% of diabetes cases [1] and has become a major public health problem worldwide [2]. Defective insulin secretion leads to type I diabetes mellitus (T1DM) and affects adolescents and children. Type II diabetes mellitus (T2DM) results from defective insulin action and affects middle- and old-aged individuals with prolonged hyperglycemia due to poor dietary choices and lifestyle [3]. T2DM is more progressive and difficult to manage [4]. T2DM is the result of the dysfunctioning of β cells of the pancreas leading either to improper utilization of insulin or its abnormal secretion because of an imbalance between insulin levels and insulin sensitivity, and hence T2DM is also referred to as “insulin resistance” [3]. Diabetic patients can develop life-threatening or serious complications. They are advised on medications to manage diabetes and to improve health [2], as body cells do not respond to insulin hormone [5]. Oral drugs are prescribed for adjunct therapy, with metformin as the first-line medication for the treatment of T2DM [6].

Metformin has minimal side effects with glucose-lowering ability, no weight gain [7], low cost, and a safety profile [8]. Metformin lowers glucose concentration by reducing insulin resistance [9], increasing glucose uptake from the periphery to skeletal muscles, enhancing insulin sensitivity, and decreasing gluconeogenesis and glucose production in the liver. The metformin mechanism of action includes the activation of protein kinase (AMPK) by 5ʹ adenosine resulting in monophosphate activation, thus leading to the oxidation of fatty acids in the liver and muscle gluconeogenesis and synthesis and inhibition of triglycerides and cholesterol. It also lowers weight and LDL cholesterol levels [7]. Compared to other pharmaceutical interventions, Metformin results in lesser hypoglycemic attacks and reduced deaths in diabetic patients.

MET has ~ 50 to 60% oral bioavailability with excellent pharmacokinetic behavior followed by absorption in the intestine. It then enters the portal vein and piles up in the liver. The excretion of the drug in urine is fixed, having a renal clearance of 510 ± 120 mL/min. Its half-life is ~ 5 h and, thus, not metabolized in the human body [6]. The recommended dose is 1–2 g/day, resulting in plasma metformin concentration from 1 to 40 μM [8]. Metformin consumed more than recommended limit can result in gastrointestinal complications like diarrhea, anorexia, and nausea [9]. Overdosage can lead to nephropathy, carcinogenicity, immunopathological conditions, bone marrow and hepatotoxicity, reproductive disorders, and allergies.

Several techniques detect metformin, i.e., GC, HPLC, NMR, IR, solid-phase extraction, CE, UV–Vis, conductometry [4], LC–MS [5], UV–Vis diffuse reflectance spectroscopy [10], and ATR–FTIR coupled with chemometrics and chemiluminescence [1]. These techniques provide valuable results [11] but have shortfalls of being sophisticated, time-consuming [1], expensive [5], large sample volume consumption [10], and complex data analysis [12]. Electrochemical methods for determining biomolecules have easier sample fabrication steps [5], fast response rate [13], accuracy, portability [1], and diverse modes of analysis [14]. Nanomaterials modify the electrodes to enhance the efficiency of electrochemical sensors. Nanoparticles enhance the surface area, electrical conductivity, catalytic effect, and optical properties [9].

For the electrochemical detection of pharmaceuticals, electroanalytical techniques such as electrochemical impedance spectroscopy (EIS), cyclic voltammetry (CV), and differential pulse voltammetry (DPV) are available [15]. CV is employed for the potentiodynamic and qualitative analysis of various analytes. It measures current by varying the applied potential of the working electrode in both anodic and cathodic sweep directions. This voltammetric technique determines stoichiometry, number of electrons transferred, reversibility, type of reaction, diffusion coefficient, activation barrier, charge transfer coefficient, and reaction kinetics. In the case of DPV, the pulse amplitude is fixed and superimposed on gradually increasing potential. The current measurement is carried out at the pulse application's start and end. Potential is calculated between these two points and is considered the potential for each pulse. Pulse width and amplitude are the parameters that optimize the signal response. EIS is an electrochemical setup for systems depicting high resistivity. Small amplitude alternating potential is applied, and response is measured. It comprises of frequency generator, frequency response analyzer (heart of EIS system), electrochemical cell, and analytes [15, 16].

Metformin monitoring uses several electrochemical sensors such as Cu(II) activated charcoal/CPE, Cu-MW/CNT/CPE, Cu(OH)_2_/CIL electrode, graphene nanoflakes-polymethylene blue/fluorine-doped tin oxide, SBA-15-Cu(II)/CPE, Cu/TiO_2_/CPE, Ɣ Fe_2_O_3_/hydroxyapatite/Cu(II)/CPE, pyrogallol/CPE, Fe–Cu/TiO_2_/CPE, BiVO_4_/CPE, Cu/polypyrrole, and Au–ZnFe_2_O_4_/CuO decorated GCE [1]. Yttrium iron garnet (YIG) is mainly a ferromagnetic transition metal compound. The involvement of iron 3d ions gives rise to optical absorption and remarkable electrical and magnetic properties [17]. Its low saturation flux density, low permeability, absorption of electromagnetic waves, and lower losses at higher frequencies enhance its application in electrical devices [18]. In addition, it has the remarkable property of reducing particle size and preventing the formation of agglomerates [19]. Cobalt doping on YIGs has attracted attention due to its domain walls, photomagnetic effect, magnetic anisotropy, and magneto-optical properties [20].

Herein, an electrochemical sensor based on cobalt-doped yttrium iron garnets is fabricated and employed to determine metformin. Comparison is made with pristine yttrium iron garnets. The electrochemical estimation of anti-diabetic metformin in PBS buffer solution and serum samples of type 2 diabetic patients is carried out. Yttrium iron garnets (doped or undoped) have never been employed to analyze drugs electrochemically. Studies are carried out to determine the role of yttrium in drug delivery; however, their potential as an electrode material has not been reported. For the first time, this article introduces yttrium-based nanoparticles as novel electrode modifiers, thus, opening up new avenues in the fabrication of electrochemical sensors.

## Experimental

### Chemicals and reagents

Yttrium (III) nitrate hexahydrate (Y_3_(NO_3_)_3_·6H_2_O), ammonium hydroxide (NH_4_OH), and cobalt nitrate (Co (NO_3_)_2_·6H_2_O) of analytical grades were purchased from Sigma-Aldrich. Iron (III) nitrate nonahydrate (Fe (NO_3_)_3_·9H_2_O) and citric acid monohydrate (C_6_H_8_O_7_·H_2_O) were procured from Daejung and Merck, respectively. All solutions were synthesized in deionized and distilled water.

### Preparation of yttrium iron garnets (YIG)

Pristine yttrium iron garnet nanoparticles were fabricated via the sol–gel method. Yttrium (III) nitrate hexahydrate and iron (III) nitrate nonahydrate were taken in stoichiometric amounts to obtain a molar concentration in 25 mL of distilled water. The citric acid solution was made by dissolving citric acid in 12.5 mL of distilled water. In a separate beaker, solutions of iron nitrate and yttrium nitrate were made by dissolving them in 12.5 mL of distilled water. The citric acid solution was added to the iron and yttrium nitrate solution. The pH was maintained at 2 by adding 2.5-mL ammonium hydroxide. The content was stirred for 4 h at 95 ℃ until gel was formed. After 48 h of shaking, the gel was heated at 150 ℃ (0.5 °C/min ramp) and for 1 h at 350 °C (1 °C/min ramp). The nanocrystalline powder was obtained by heating at 900 ℃ for 2 h.

$${\text{Y}}\left( {{\text{NO}}_{{3}} } \right)_{{3}} \cdot{\text{6H}}_{{2}} {\text{O}} + {\text{Fe}}\left( {{\text{NO}}_{{3}} } \right)_{{3}} \cdot{\text{9H}}_{{2}} {\text{O}} \to {\text{Y}}_{{3}} {\text{Fe}}_{{5}} {\text{O}}_{{{12}}}$$

### Preparation of cobalt-doped yttrium iron garnets (Co-doped YIG)

Cobalt-doped YIG was synthesized similarly as above. There was a change in the first step, where cobalt nitrate was added along with citric acid in the first beaker, and 12.5-mL distilled water was added to form the solution. The remaining steps were performed in the same order as reported in a previous study [20].

$${\text{Y}}\left( {{\text{NO}}_{{3}} } \right)_{{3}}\cdot {\text{6H}}_{{2}} {\text{O}} + {\text{Fe }}\left( {{\text{NO}}_{{3}} } \right)_{{3}} \cdot{\text{9H}}_{{2}} {\text{O}} + {\text{Co}}\left( {{\text{NO}}_{{3}} } \right)_{{2}} \cdot{\text{6H}}_{{2}} {\text{O}} \to {\text{Y}}_{{3}} {\text{Fe}}_{{5}} {\text{Co}}_{x} {\text{O}}_{12}$$

### Preparation of working electrode

A glassy carbon electrode (GCE) was polished with polishing cloth and alumina powder suspension to remove the contaminants and activate the electrode surface. The electrode was further washed with distilled water using a sonicator. 5-μL suspension (pristine YIG and cobalt-doped YIG mixed in deionized water separately) was deposited on GCE and dried at ambient temperature for 15 min to attach pristine- and Co-doped YIG successfully. The fabricated electrodes were ready for use with a lifespan of ~ a few weeks.

### Electrochemical analysis of metformin

Potentiostat (PG-STAT) PGSTAT302N Metrohm Switzerland was employed to determine metformin in PBS and serum samples of T2DM via the voltammetric or electrochemical cell having reference (Ag/AgCl), working (glassy carbon electrode), and counter electrodes (platinum wire). The behaviors of bare, undoped, and cobalt-doped YIG-modified GCE were analyzed by the cyclic voltammetry. Metformin solutions of varied pH and concentrations were analyzed via differential pulse voltammetry. All measurements were made at room temperature.

### Serum sample collection and processing

Samples of diabetic patients were taken for electrochemical analysis of metformin by fabricated nanoparticles. Samples were collected in serum collection tubes after patients' informed consent and according to the guidelines and approval of the Ethical Committee of Sahiwal Medical College, Sahiwal, Pakistan. The blood samples were centrifuged at 4000 rpm for 10 min to obtain serum samples of T2DM patients.

## Results and discussion

### Characterization of YIG and Co-doped YIG

**Fig. 1:**
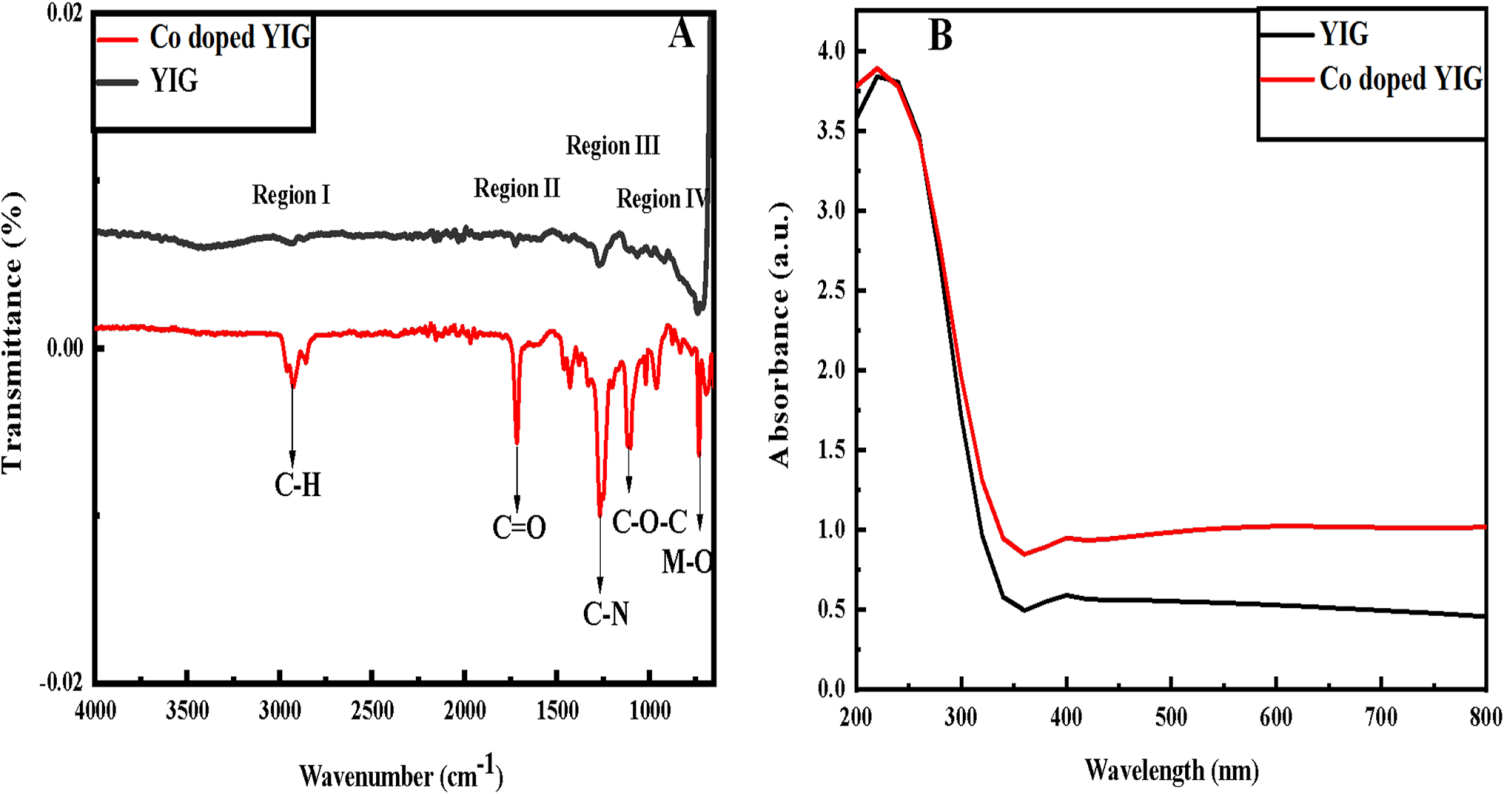
**A** FT-IR, and **B** UV Spectra of pristine and Co-doped YIG

Fourier transmission infrared spectroscopy (FTIR) spectra are obtained by measuring the transmittance from 4000 to 650 cm^−1^ on INVENIO FTIR Spectrometer Bruker Germany. FTIR spectra of pristine and Co-doped YIG sintered at 900 °C for 2 h are shown in Fig. 1A.

IR spectra were divided into regions for the interpretation of functionalities. In region I, the peak at 2929 cm^−1^ appears for both pristine YIG and Co-doped YIG representing the C–H stretch. Region II depicts a peak at 1714 cm^−1^ of C = O. Region III comprises peaks at 1266 cm^−1^ and 1103 cm^−1^ for C–N and C–O–C, respectively [21]. The bands from 800 to 400 cm^−1^ depict vibrations of metal oxide in garnet. A peak at 733 cm^−1^ shows Fe–O and Co–O in YIG and Co-doped YIG, respectively. The electronegativity of cobalt is more than that of iron, which enhances the damping and alleviates the vibrational intensities of smaller wavelengths [22]. Ramesh et al. reported that the stretching vibration of Y–O in YIG can appear around 300 cm^−1^ which is beyond our measured range [23]. The peaks for doped material have a higher intensity than pristine nanomaterial.

UV–Vis analysis is performed to study the absorptions of pristine and cobalt-doped YIG via AQ7100APAC Thermo Fischer Scientific UK Spectrophotometer. Figure 1B illustrates the absorption spectra of prepared materials in the 200–800-nm range. The band is 220 nm in both pristine and cobalt-doped YIG. The literature reports that rare earth metals, i.e., yttrium give absorption at the wavelength range from 220 to 450 nm [24]. Band gap energy is calculated by the mentioned equation:

$$E = \frac{1240}{{\lambda \,\left( {{\text{nm}}} \right)}}\,{\text{eV}}$$

The observed wavelengths are 339 nm and 341 nm for Co-doped YIG and pristine YIG, respectively. The calculated band gap is 3.65 eV and 3.63 eV for Co-doped YIG and pristine YIG, respectively. No significant change is observed in the band gap.

**Fig. 2:**
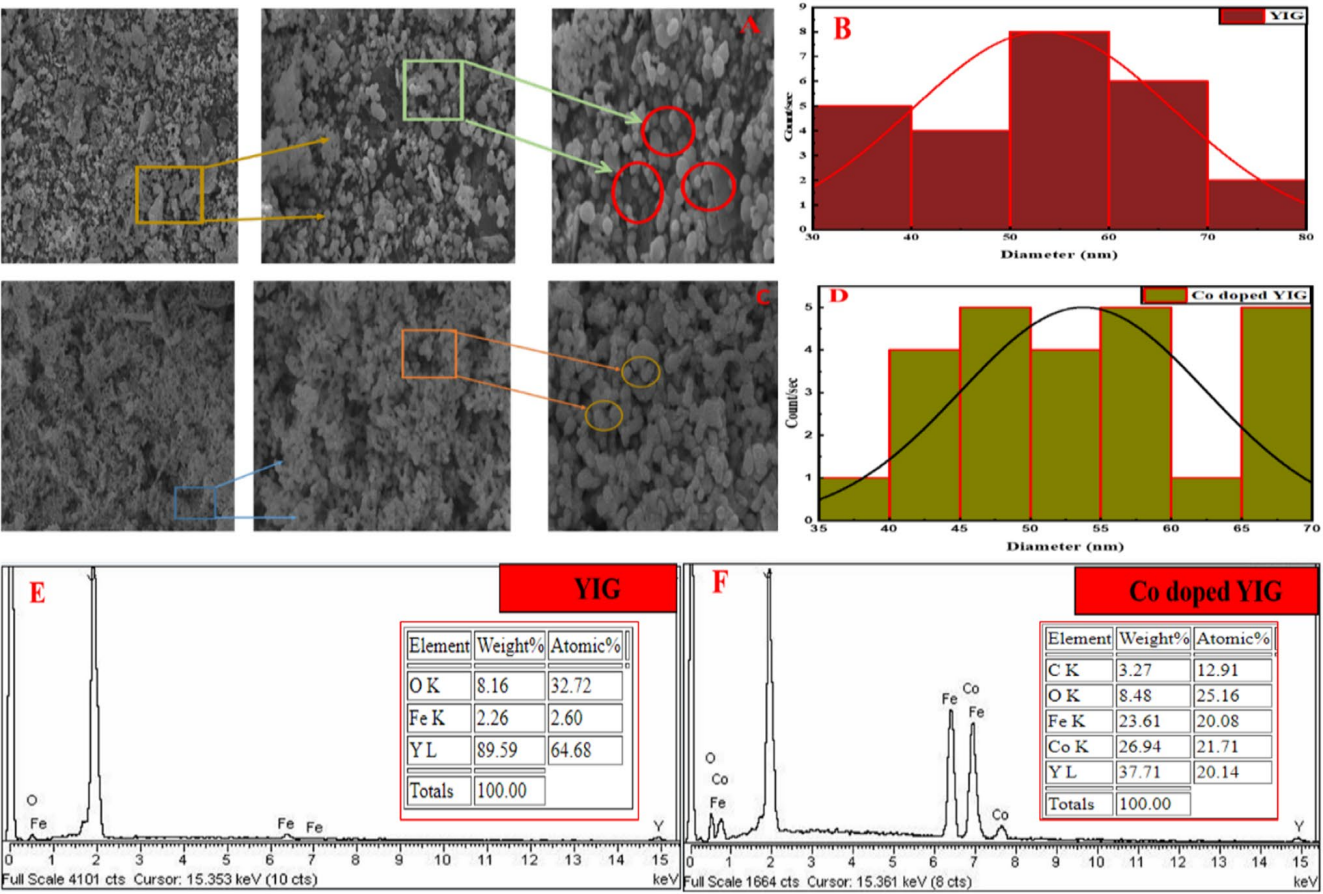
**A**, **C** SEM images of YIG and Co-doped YIG, **B**, **D** Histograms representing particle size distribution of YIG and Co-doped YIG, and **E**, **F** EDX of YIG and Co-doped YIG

SEM images (JSM-7200F JEOL Japan) evaluate the morphology and size of undoped and Co-doped YIG (Fig. 2). SEM images show that the fabricated material is asymmetrical with different sizes and shapes. The histogram in Fig. 2B reveals the particle size distribution of YIG developed via SEM image data. Particles are predominantly 30–80 nm, with an average diameter of around 53 nm. Figure 2D depicts the histogram of Co-doped YIG where particles range from 35 to 70 nm with an average diameter of around 54 nm.

EDX analysis provides an elemental composition of YIGs. EDX graphs of pristine and Co-doped YIG are given in Fig. 2E, F, showing all the elements of both YIGs. Figure 2E illustrates the wide scan of YIG and depicts oxygen, iron, and yttrium peaks. No cobalt or any other element is detected in pristine YIG particles. Figure 2F is the EDX spectrum of Co-doped YIG showing iron, cobalt, oxygen, and yttrium with no impurities.

**Fig. 3:**
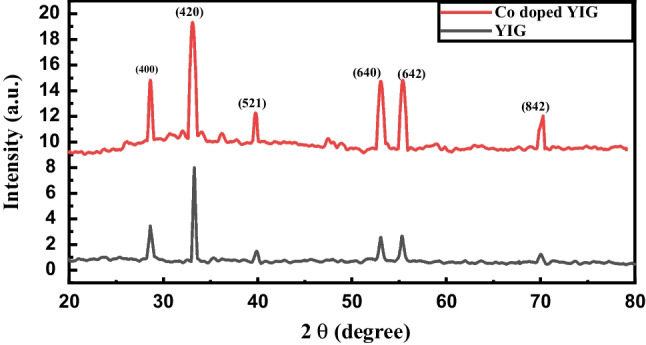
XRD spectrum of YIG and, Co-doped YIG

XRD confirms the phase formation in doped and undoped materials. The sample shows a single phase confirmed by reference code (COD 96-100-8629). Reference code belongs to the structure of YIGs and does not involve any other secondary phase regardless of Co concentration. XRD analyses of doped and undoped YIGs on Bruker D8 Advance Powder X-ray Diffractometer (Bruker, Germany) are given in Fig. 3. The assigned 2θ values for undoped and doped YIG for (400), (420), (521), (640), (642), and (842) planes are 28.5̊, 33.2̊, 39.8̊, 53.0̊, 55.3,° and 70.0̊, respectively. These peaks are attributed to the planes of a cubic unit cell. The sharp peaks depict the crystalline nature of fabricated materials. Peaks at (400) and (420) affirm that synthesized YIGs belong to the Ia3d space group with the cubic crystal structure. The presence of the garnet phase for YIG is confirmed via the structural peak at (420) [24].

Crystallite size is calculated through the Scherrer formula:

$$D = 0.9\frac{\lambda }{\beta } \times \cos \theta$$

where *λ* indicates wavelength, *β* is the width at half of the maximum peak intensity, and *θ* denotes incident ray angle. The crystallite size of YIG and Co-doped YIG from the Scherrer formula is 21.2 nm and 17.2 nm, respectively.

### Evaluation of GCE/YIG and GCE/Co-doped YIG

Cyclic voltammetry confirms the immobilization and studies the electrochemical characteristics of fabricated material on GCE in 0.1 M KCl and 0.1 mM K_4_[Fe(CN)_6_]^3−/4−^ solutions.

**Fig. 4:**
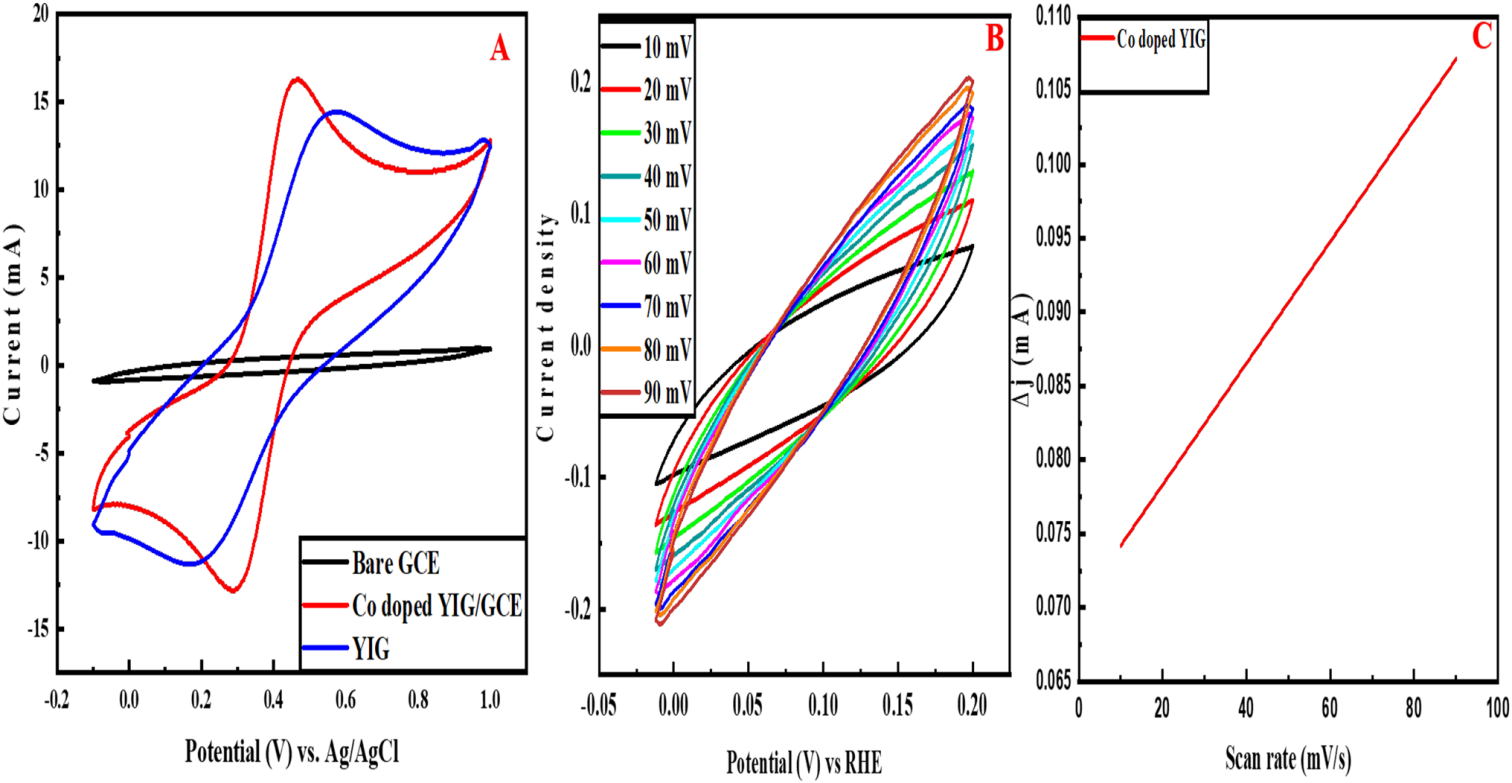
**A** Cyclic voltammogram depicting electrical conductivity of bare, doped, and undoped yttrium iron garnet particles. **B** Cyclic voltammogram depicting ECSA of Co-doped YIG on GCE at varying scan rates in 0.1 M potassium ferricyanide containing 0.1 M KCl, and **C** Corresponding calibration graph of ECSA

The results depict that no oxidation–reduction takes place on bare GCE. Both doped and undoped materials show the redox phenomenon. Figure 4A shows pure and cobalt-doped YIG conductivity peaks. Cyclic voltammogram exhibits that doping enhances the conductance of YIG. An increase in peak current is observed in Co-doped YIG attributed to doping with cobalt. The elevated number of free electrons due to doping increases the conductivity. Therefore, Co-doped YIG is preferred over YIG in further analysis of metformin.

### Electrochemical active surface area (ECSA) of Co-doped YIG

Co-doped YIG is assessed by using a 1:1 M ratio of KCl (0.1 M) and [Fe (CN)_6_]^3−/4−^ (0.1 M). CV analysis is performed in the presence of electrolyte solution at scan rates from 10 to 90 mV/s, as shown in Fig. 4B. ECSA of Co-doped YIG/GCE and bare GCE are calculated by Randles–Sevcik equation, and the values are 0.205 cm^2^ and 0.073 cm^2^, respectively. The ECSA values are obtained using the line graph depicted in Fig. 4C. According to these findings, the synthesized nanoparticles lead to an elevated surface area of GCE and prove to be an efficient platform depicting high supramolecular recognition capabilities with raising conductivity.

### Mechanism of electrochemical sensing of metformin

**Fig. 5:**
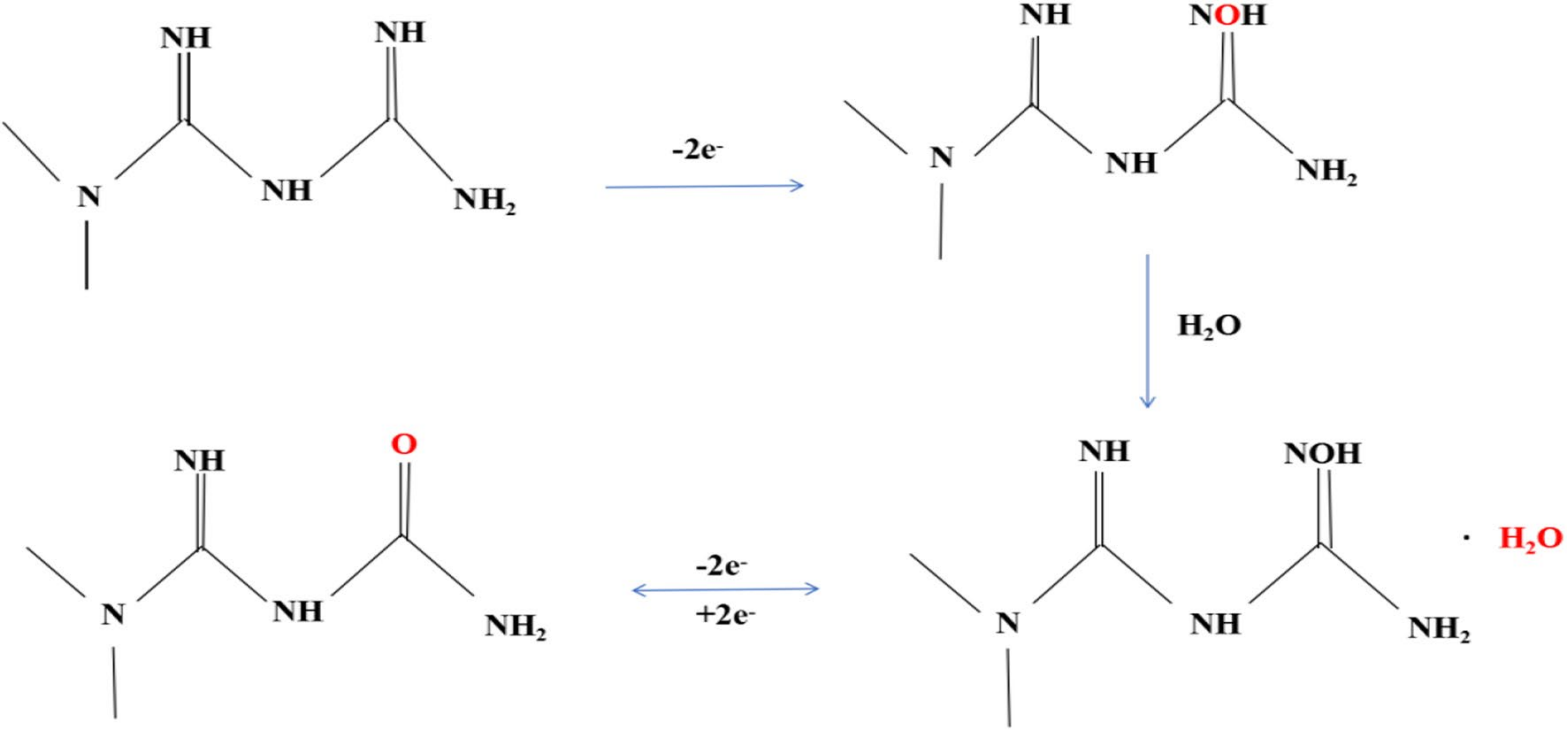
Electrochemical oxidation of metformin

Metformin oxidation at the electrode leads to an oxidation peak. This results from the electrochemical oxidation of imino group (present in guanidino compounds) of metformin to *N*-hydroxyimino group that gets hydrolyzed to the carbonyl imino group [9, 10]. The complete mechanism is given in Fig. 5.

Co-doped YIG is fabricated to assist in the electron mentioned above transfer mechanism and functions as an electrochemical transducer for metformin detection via DPV.

### Optimization of differential pulse voltammetric (DPV) parameters

**Fig. 6:**
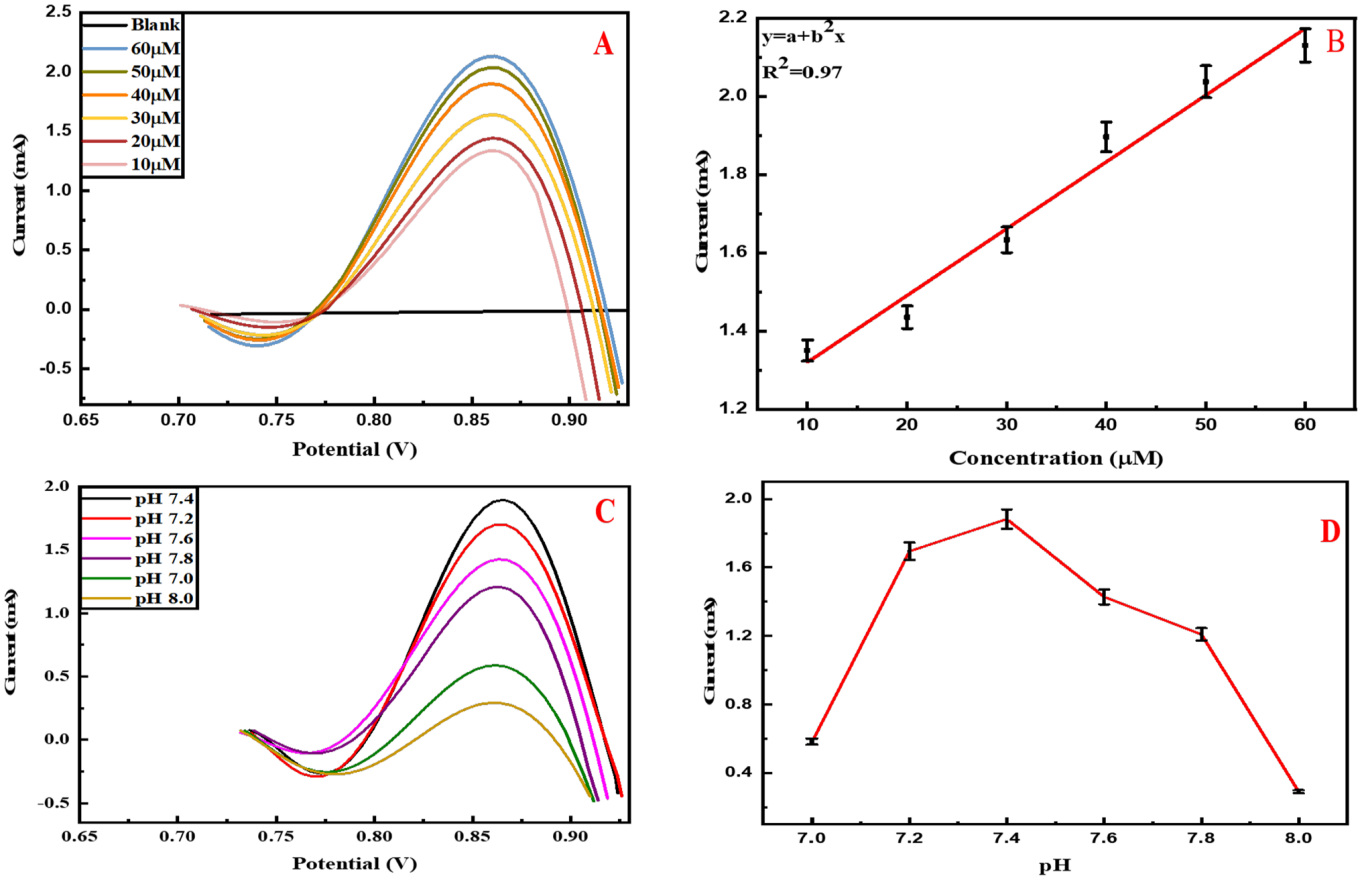
**A** Differential pulse voltammogram depicting the effect of varying concentrations, **C** effect of varying pH 7.0–8.0 on electrochemical response of Co-doped YIG for metformin detection in 0.1 M PBS solution of pH 7.4, **B** line graph at varying metformin concentrations, and **D** Calibration plot at varying pH

DPV analyzes the effect of various concentrations of metformin on Co-doped YIG. No behavior is observed in the blank PBS solution. There is an increase in oxidation peak current by increasing metformin concentration. The maximum peak current is at 60 μM and the lowest at 10 μM metformin concentration in PBS (0.1 M) solution of pH 7.4 (Fig. 6A). A linearity (R2) of 0.97 is obtained as shown in Fig. 6B, representing the direct relationship between current and metformin concentration. Similarly, the effect of varied pH (7.0, 7.2, 7.4, 7.6, 7.8, and 8.0) is observed via DPV, as presented in Fig. 6C. The maximum peak is observed at pH 7.4, and the lowest at 8.0, and the calibration plot is given in Fig. 6D.

**Fig. 7:**
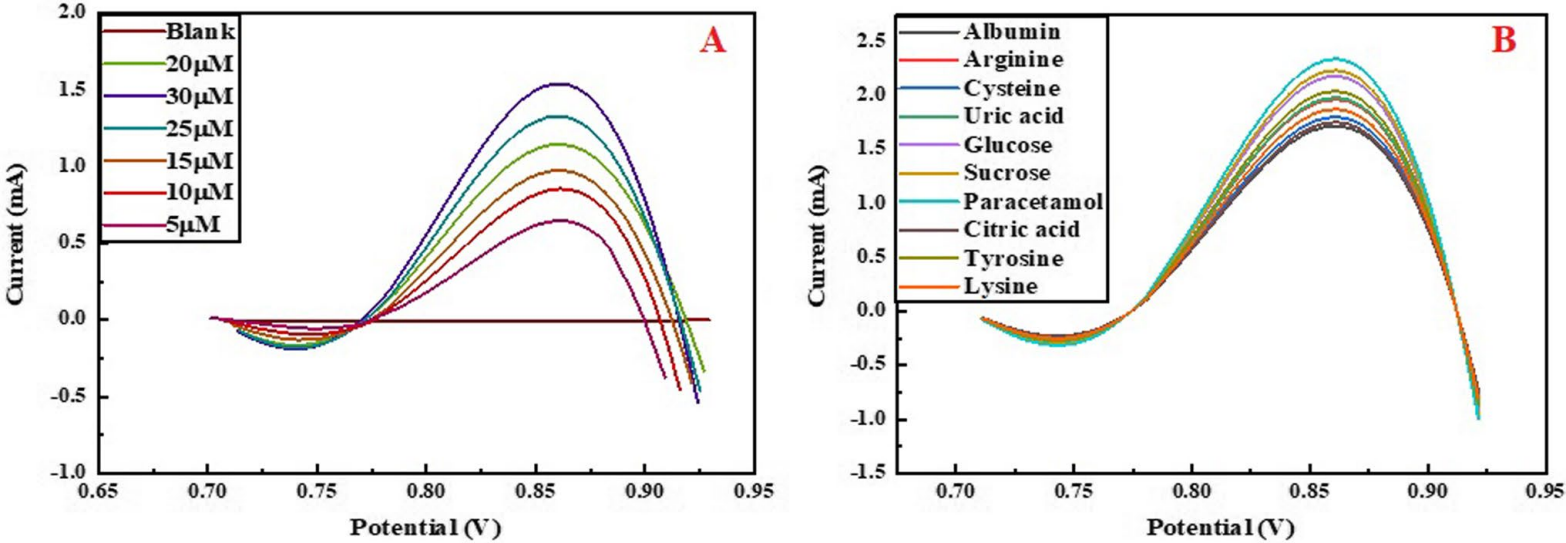
**A** Differential pulse voltammogram metformin at lower concentrations (5–20 µM) and **B** effect of different interefering species in metformin detection

The analytical investigation of cobalt-doped yttrium iron garnet is also performed at lower concentrations of metformin (5 µM to 20 µM). Thus, obtained differential pulse voltammogram depicts that the fabricated sensor detects the metformin at a lower concentration (Fig. 7A). Moreover, the effect of interfering species (paracetamol, citric acid, glucose, albumin, arginine, lysine, tyrosine, uric acid, cysteine, and sucrose) is also investigated on metformin detection (60 µM) by Co-doped YIG. Obtained DPV curves suggest that these interfering species do not affect the sensing performance of the material (Fig. 7B).

### Electrochemical impedance spectroscopic (EIS) analysis

**Fig. 8:**
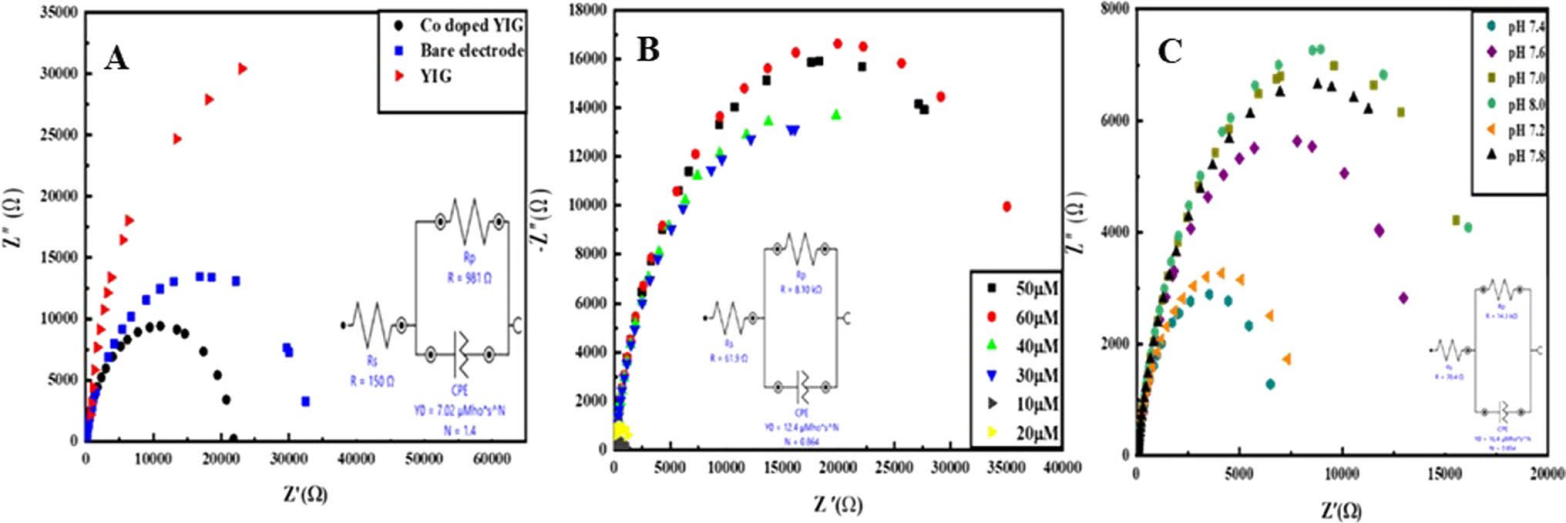
Electrochemical impedance graph of **A** Bare GCE, undoped YIG, and Co-doped YIG, **B** at varying metformin concentrations, and **C** at various pH of 60 µM metformin concentration

Another powerful technique for the analysis of electrode modification is electrochemical impedance spectroscopy (EIS), which is utilized to evaluate electrode response at different concentrations and pH. Charge transfer resistance (*R*_ct_) is used to study the probe kinetics at the electrode surface. This parameter deals with substrate immobilization on the modified electrode. Nyquist curves in the presence of a redox probe vs. Ag/AgCl/3 M KCl are plotted to analyze the *R*_ct_ parameter. Figure 8A illustrates the Nyquist diagram for different electrodes in the presence of K_4_[Fe (CN)_6_] (0.1 mM) and KCl (0.1 M).

The Nyquist plot was fitted by Randles equivalent circuit to attain quantitative data from impedance data. From this fitting, the obtained *R*_ct_ for the bare electrode is 6.93 kΩ which is smaller than the *R*_ct_ of YIG/GCE (8 kΩ). *R*_ct_ of YIG/GCE is larger than Co-doped YIG/GCE (981 Ω), thus indicating that doping with cobalt increases the electrical conductivity of YIG [25, 26]. The results show that Co-doped YIG has better sensing abilities than pristine YIGs.

The effect of concentration on impedance is shown in Fig. 8B. An increased metformin concentration decreases the interfacial electron transfer resistance on the electrode surface affected by electrostatic interactions and is proportional to analyte detection. Impedance observed at various pH (7.0, 7.2, 7.4, 7.6, 7.8, 8.0) is shown in Fig. 8C. Impedance is the lowest at pH 7.4, and *R*_ct_ values shift by varying the pH of the solution.

### Heterogeneous electron transfer rate constant (*k*°)

Electrochemical impedance spectroscopy (EIS) is applied to analyze charge transfer at the electrode surface. The electrochemical impedance behavior is checked at various electrodes in the same circuit. The circuit comprises electron transfer resistance (Rct), Warburg impedance (*Z*_w_), interfacial capacitance (Cdl), and electrolyte ohmic resistance (*R*_s_). In EIS, linear as well as semi-circular segments are evaluated. At the electrode interface, the semicircle diameter measures the kinetics of electron transfer of the redox probe and detects *R*_ct_. Linear sections correspond to diffusion at lower frequencies. *R*_ct_ of Co-doped YIG is 981 Ω, and of bare GCE is 6.93 kΩ.

Co-doped YIG functions by increasing the rate of electron transfer. A reciprocal correlation is found between surface resistance and electrical conductivity. It is proved from electrochemical parameters that Co-doped YIG-deposited GCE has better electron transfer kinetics than the bare electrode. This is attributed to the larger surface area of Co-doped YIG.

EIS technique evaluates the standard heterogeneous rate constant (*k*°) [27].

$$k^\circ = \frac{RT}{{F_{2} R_{{{\text{ct}}}} AC}}$$

*R* is the gas constant (8.314 J K^−1^ mol^−1^), *T* is the thermodynamic temperature (298.15 K), F is Faraday constant (96,485 C mol^−1^), *R*_ct_ is electron transfer resistance (6.93 kΩ and 981 Ω for bare and Co-doped YIG, respectively), *A* is electrode surface area (0.073 cm^2^ of bare GCE and 0.205 cm^2^ for Co-doped YIG), and *C* is the concentration of [Fe(CN)_6_]^3−/4−^ solution. The unit for *k*° is cm s^−1^ and represents the standard heterogeneous rate constant. The obtained *k*° for bare GCE and Co-doped YIG/GCE is 5.2 × 10^−6^ cm s^−1^ and 1.3 × 10^−5^ cm s^−1^, respectively. Systems giving higher *k*° values establish equilibrium much faster, indicating enhanced electron transfer.

### Roughness factor (Rf)

The roughness factor is obtained by calculating the peak current (*I*_pa_) of [Fe (CN)_6_]^3−/4−^ through the redox couple that corresponds to blank GCE. The factor depends on the number of oxidation–reduction cores as specified by the electrochemical procedure, and the cores depend on the electrode surface and dimension [28]. The Rf factor depends on the two electrodes' oxidation peak current and surface areas. The current ratio for both electrodes illustrates changes in the surface area of the electrode. The equation is as follows:

$$R_{{\text{f}}} = \frac{{Ip^{2} }}{{Ip^{1} }} = \frac{{A_{2} }}{{A_{1} }}$$

*Ip*1 and *Ip*2 represent peak currents for bare and co-doped YIG-modified GCE. The bare (*A*_1_) and modified GCE (*A*_2_) surface areas are 0.073 cm^2^ and 0.205 cm^2^, respectively. The calculated Rf value is 2.80.

### Interference

**Fig. 9:**
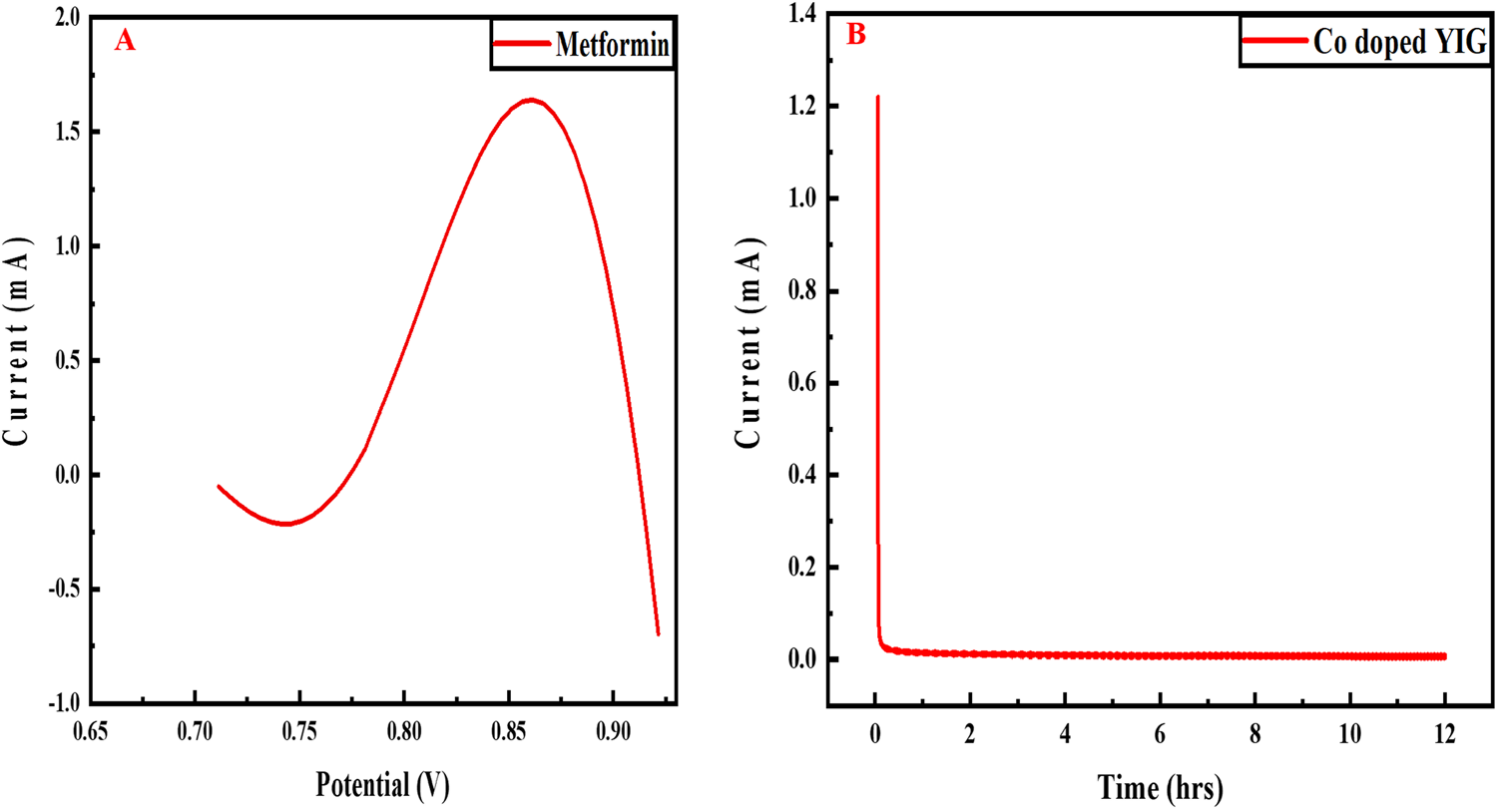
**A** Interference plot for metformin solution with interfering agents, **B** chronoamperometric graph of Co-doped YIG in the presence of redox probe

To check the selective measuring of metformin in the presence of different species, interference studies were performed. The interfering species, i.e., paracetamol, citric acid, glucose, albumin, arginine, lysine, tyrosine, uric acid, cysteine, and sucrose, are added in 60-µM metformin solution to test the selectivity of fabricated material toward metformin, and the resulting response is shown in Fig. 9A. The interfering substances are added in the same and doubled concentration to metformin, but the oxidation peak for metformin remains prominent. This indicates the selective sensing response of Co-doped YIG in the presence of interferences.

### Stability of Co-doped YIG/GCE

The durability and steady-state activity of the material are evaluated through chronoamperometry at 0.01 V for 12 h, and the obtained curve for Co-doped YIG/GCE is shown in Fig. 9B. The peak illustrates the electrochemical behavior and stability of Co-doped YIG nanocomposite.

### Evaluation of limit of detection (LOD)

LOD represents the lowest quantity of analyte detected, which is specific for an analyte and influenced by factors like buffer condition, matrix, and technique employed. The following equation determines it:

$${\text{LOD}} = 3\frac{s}{m}$$

where *s* denotes the standard deviation and *m* is the slope. The calculated LOD is 0.04 μM.

### Recovery analysis of metformin by Co-doped YIG

Co-doped YIG's applicability to serum samples is tested by measuring the recovery of metformin in serum samples of T2DM. Serum samples are diluted 10 times with PBS of pH 7.4. Metformin recovery is examined by utilizing different concentrations of standard metformin. The results are shown in Table 1, where recovery ranges from 83.6 to 95%. The recovery results show the potential of Co-doped YIG for metformin detection in the serum of diabetic patients. The obtained recovery indicates the accuracy of the fabricated methodology.

**Table 1:** Metformin recovery studies on Co-doped YIG from serum samples

Samples	Added conc (µM)	Found conc (µM)	Recovery (%)
S1	60	54.1	90.1
S2	60	56.0	93.3
S3	60	57.0	95
S4	60	50.2	83.6

### Metformin detection in the serum samples of T2DM patients

**Fig. 10:**
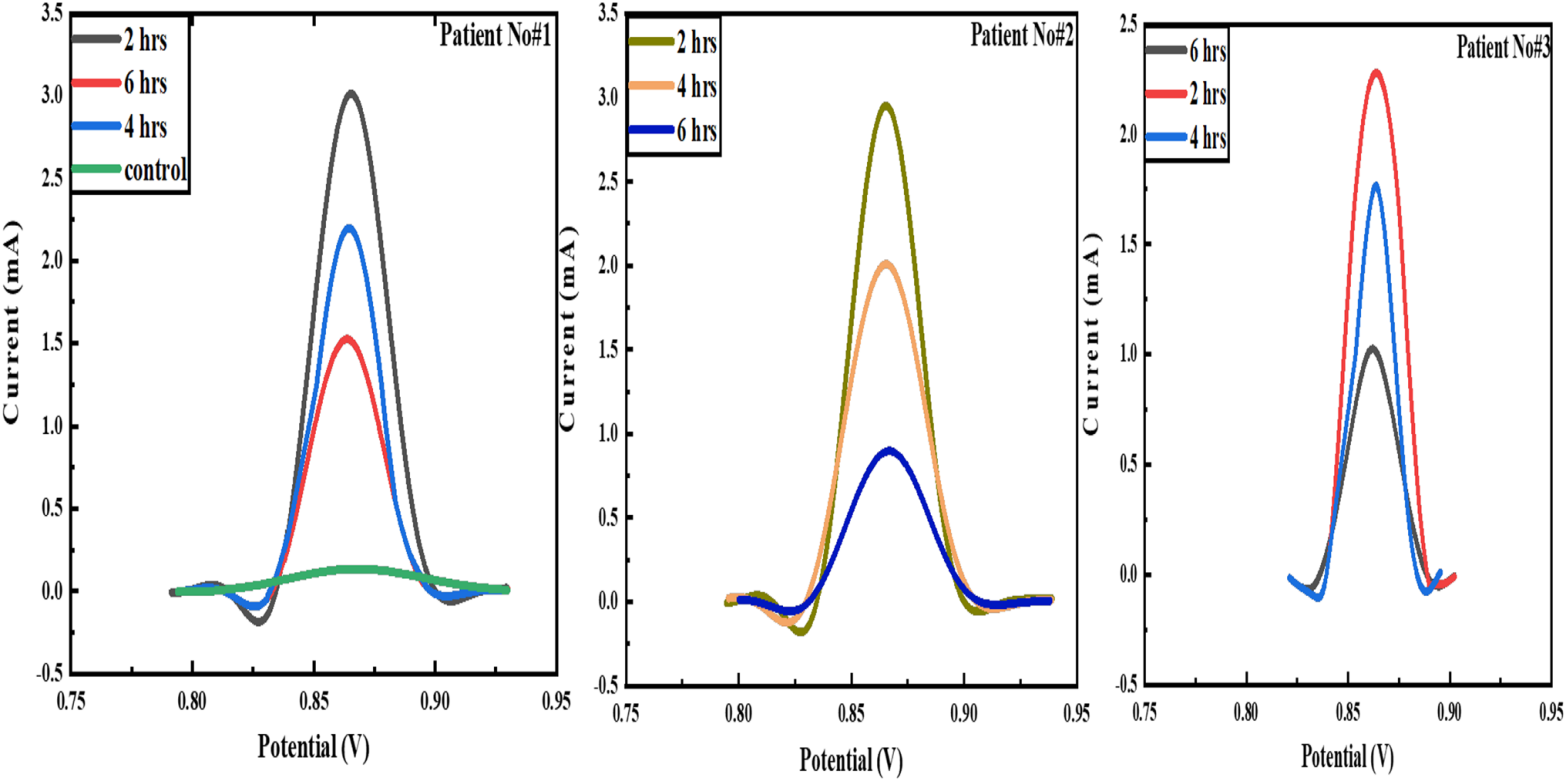
Metformin detection in serum samples of T2DM patients at different time intervals via Co-doped YIG-modified GCE

Metformin reaches its half-life in 2–6 h, where the drug concentration reduces by 50% in the human body. The potential of the fabricated sensor for metformin determination in T2DM patients is found at different times. The serum of diabetic patients who took the metformin dose is analyzed. Blood samples are taken after 2, 4, and 6 h of metformin intake. As shown in Fig. 10, the three patients show a sharp peak during the initial 1–3 h, illustrating that metformin concentration is the highest in the body and reduces with time. This indicates the reproducibility of the developed sensor where three patients show similar behavior. A little variation in current is observed, which is natural as metabolic rates vary from individual to individual.

A comparison of fabricated Co-doped YIG sensors with previously reported material is given in Table 2.

**Table 2:** Sensing modalities, LODs, and linear ranges of recently employed sensors for metformin detection

Sr. no	Sensor	Sensing modality	Linear range (μM)	LOD (μM)	Reference
1	CB-RuO_2_-Nafion/GCE	ASDPV	0–70	0.7	[5]
2	ZnFe_2_O_4_/CuO/GCE	CV, DPV	1 nM–1 µM	0.3	[4]
3	SBA-15-Cu (II)/CPE	DPV	65–0.1	0.03	[29]
4	GNF-PMB/SnO_2_/F	CV	100–1000	0.00001	[30]
5	Ɣ-Fe_2_O_3_@HAp/Cu(II)/CPE	ASDPV	0.1–80	0.014	[10]
6	Co-doped YIG/GCE	DPV	0–60	0.04	Current work

## Conclusion

A sensitive and selective electrochemical sensor is developed for the quantitative analysis of metformin. For the first time, Co-doped YIG prepared via the sol–gel method is used for metformin detection. The morphological and structural results confirm the formation of pristine YIG and Co-doped YIG. Best electroanalytical results are obtained at 60-μM metformin concentration at pH 7.4 with LOD 0.04 μM. MET redox strategy is simple and does not perplex the fabrication method. The modifications at the electrode surface enhance the electrode’s performance. The developed electrochemical sensor is facile and low-cost for determining metformin in pharmaceutical and real clinical samples. The sensor can monitor metformin in drug delivery systems. The fabricated detection methodology can be utilized in the pharmaceutical industry for quality control.

## Data Availability

All data generated or analyzed during this study are included in this article.
